# P-611. Time-Series Model Estimation of Respiratory Syncytial Virus-Attributable Respiratory Hospitalizations and Mortality in Adults in Finland

**DOI:** 10.1093/ofid/ofaf695.824

**Published:** 2026-01-11

**Authors:** Aleksandra Polkowska-Kramek, Robin Bruyndonckx, Caihua Liang, Mikko Kosunen, Olli-Pekka Hätinen, Mikel Esnaola, Maribel Casas, Worku Biyadgie Ewnetu, Pimnara Peerawaranun, Bradford D Gessner, Elizabeth Begier

**Affiliations:** P95, Leuven, Brabant Wallon, Belgium; P95, Leuven, Brabant Wallon, Belgium; Pfizer Inc, New York, NY; Pfizer Oy, Helsinki, Uusimaa, Finland; Pfizer Oy, Helsinki, Uusimaa, Finland; P95 Clinical and Epidemiology Services, Llinars del Vallès, Catalonia, Spain; P95, Leuven, Brabant Wallon, Belgium; P95, Leuven, Brabant Wallon, Belgium; P95, Leuven, Brabant Wallon, Belgium; EpiVac Consulting, Anchorage, Alaska; Pfizer Vaccines, Dublin, Dublin, Ireland

## Abstract

**Background:**

As in other countries, respiratory syncytial virus (RSV) incidence among adults in Finland is still underreported mostly due to non-specific RSV symptoms, infrequent standard-of-care testing, and reduced sensitivity of single-specimen nasal/nasopharyngeal RT-PCR testing among adults. We retrospectively estimated RSV-attributable incidence of hospitalizations and mortality in adults in Finland between 2011–2019.

**Figure:**
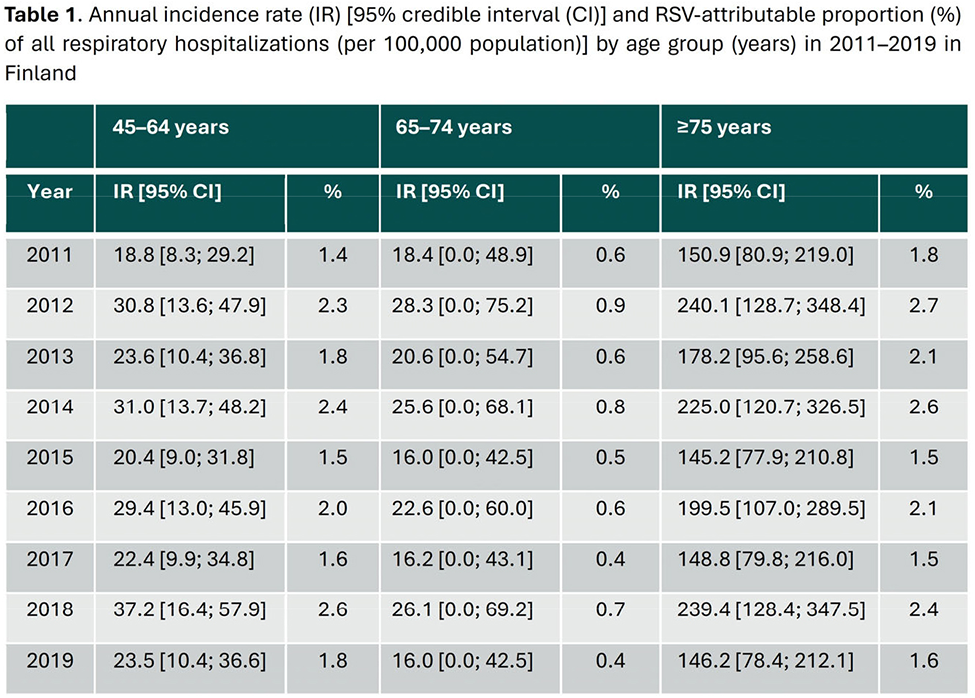

**Figure:**
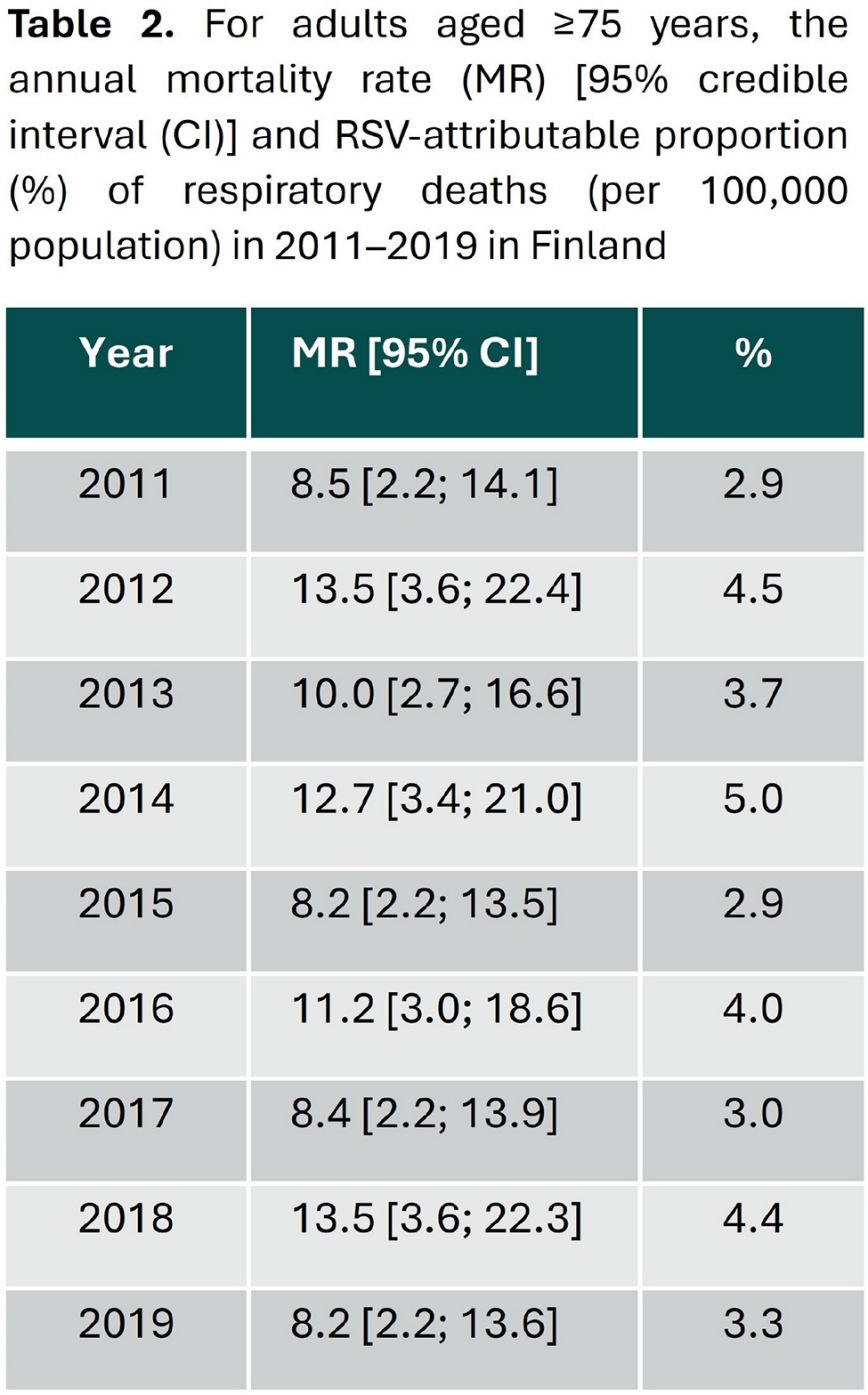

**Methods:**

We estimated incidence using time-series modeling by comparing the week-to-week variability in RSV diagnosis trends with the week-to-week variability in the events with any respiratory diagnosis. Weekly aggregated data on respiratory hospitalizations and deaths (J00–J99) were obtained from the Care Register for Health Care (HILMO) and Statistics Finland, respectively. Hospitalization data on RSV in children aged < 2 years and influenza in adults aged ≥65 years, representing viral circulation in the modeling, were obtained from HILMO. Data for age groups that showed a seasonal respiratory disease pattern were included in a hierarchical Bayesian model, sharing information across the age groups while accounting for seasonal fluctuations, and RSV and influenza circulation.

**Results:**

The highest annual incidence rates of RSV-attributable respiratory hospitalizations were observed in adults aged ≥75 years (range 145-240 hospitalizations per 100,000 person-years), on average 7-fold higher than in adults aged 45–64 years (range 19-37 hospitalizations per 100,000 population) (Table 1). A strong bi-annual (low-high incidence) fluctuation of respiratory hospitalization incidence and mortality rate were observed in all age groups. A seasonal pattern for respiratory deaths was observed only for adults aged ≥75 years, who had an estimated RSV-attributable mortality rate of 8–14 deaths per 100,000 population (Table 2). RSV-attributable deaths accounted for 3–5% of all respiratory deaths in this age group.

**Conclusion:**

Our study emphasizes the substantial respiratory morbidity and mortality associated with RSV infection among adults in Finland, particularly those aged ≥75 years. The newly introduced RSV vaccines, which appear effective for the oldest adults, could have a substantial impact on this respiratory disease burden.

**Disclosures:**

Aleksandra Polkowska-Kramek, n/a, Pfizer: Grant/Research Support Caihua Liang, MD, PhD, Pfizer: I am an employee.|Pfizer: Stocks/Bonds (Public Company)|Pfizer: Stocks/Bonds (Public Company) Mikko Kosunen, M.Sc. (Health Economics), Pfizer: Employee|Pfizer: Stocks/Bonds (Public Company) Olli-Pekka Hätinen, PhD, Pfizer: Employee|Pfizer: Stocks/Bonds (Public Company) Bradford D. Gessner, MD, MPH, Pfizer: Stocks/Bonds (Public Company) Elizabeth Begier, MD, M.P.H., Pfizer: I am an employee.|Pfizer: Stocks/Bonds (Public Company)

